# Nano-pesticidal potential of *Cassia fistula* (L.) leaf synthesized silver nanoparticles (Ag@*Cf*L-NPs): Deciphering the phytopathogenic inhibition and growth augmentation in *Solanum lycopersicum* (L.)

**DOI:** 10.3389/fmicb.2022.985852

**Published:** 2022-08-26

**Authors:** Mohammad Danish, Mohammad Shahid, Lukman Ahamad, Kashif Raees, Ashraf Atef Hatamleh, Munirah Abdullah Al-Dosary, Abdullah Mohamed, Yasmeen Abdulrhman Al-Wasel, Udai B. Singh, Subhan Danish

**Affiliations:** ^1^Section of Plant Pathology and Nematology, Department of Botany, Aligarh Muslim University, Aligarh, India; ^2^Department of Agricultural Microbiology, Faculty of Agricultural Sciences, Aligarh Muslim University, Aligarh, India; ^3^Plant-Microbe Interaction and Rhizosphere Biology Lab, ICAR-NBAIM, Mau, India; ^4^Department of Chemistry, Chandigarh University, Mohali, India; ^5^Department of Botany and Microbiology, College of Science, King Saud University, Riyadh, Saudi Arabia; ^6^Research Centre, Future University in Egypt, New Cairo, Egypt; ^7^Hainan Key Laboratory for Sustainable Utilization of Tropical Bioresource, College of Tropical Crops, Hainan University, Haikou, China

**Keywords:** *Cassia fistula*, silver nanoparticles (Ag@*Cf*L-NPs), phytopathogens, nano-pesticides, tomatoes (*Solanum lycopersicum* L.), antioxidant enzymes

## Abstract

Plant-based synthesis of silver nanoparticles (Ag-NPs) has emerged as a potential alternative to traditional chemical synthesis methods. In this context, the aim of the present study was to synthesize Ag-NPs from *Cassia fistula* (L.) leaf extract and to evaluate their nano-pesticidal potential against major phyto-pathogens of tomato. From the data, it was found that particle size of spherical *C. fistula* leaf synthesized (Ag@*Cf*L-NPs) varied from 10 to 20 nm, with the average diameter of 16 nm. Ag@*Cf*L-NPs were validated and characterized by UV-visible spectroscopy (surface resonance peak λ_*max*_ = 430 nm), energy dispersive spectrophotometer (EDX), Fourier transform infrared (FTIR), and X-ray diffraction pattern (XRD), and electron microscopy; scanning electron microscopy (SEM), and transmission electron microscopy (TEM). The FTIR spectra verified the participation of various living molecules (aromatic/aliphatic moieties and proteins) in synthesized Ag@*Cf*L-NPs. The anti-phytopathogenic potential of Ag@*Cf*L-NPs was assessed under *in vitro* conditions. Increasing doses of Ag@*Cf*L-NPs exhibited an inhibitory effect against bacterial pathogen *Pseudomonas syringae* and 400 μg Ag@*Cf*L-NPs ml^–1^ caused a reduction in cellular viability, altered bacterial morphology, and caused cellular death Furthermore, Ag@*Cf*L-NPs reduced exopolysaccharides (EPS) production and biofilm formation by *P. syringae* Additionally, Ag@*Cf*L-NPs showed pronounced antifungal activity against major fungal pathogens. At 400 μg Ag@*Cf*L-NPs ml^–1^, sensitivity of tested fungi followed the order: *Fusarium oxysporum* (76%) > *R. solani* (65%) > *Sarocladium* (39%). Furthermore, 400 μg Ag@*Cf*L-NPs ml^–1^ inhibited the egg-hatching and increased larval mortality of *Meloidogyne incognita* by 82 and 65%, respectively, over control. Moreover, pot studies were performed to assess the efficacy of Ag@*Cf*L-NPs to phyto-pathogens using tomato (*Solanum lycopersicum* L.) as a model crop. The applied phyto-pathogens suppressed the biological, physiological, and oxidative-stress responsiveness of tomatoes. However, 100 mg Ag@*Cf*L-NPs kg^–1^ improved overall performance and dramatically increased the root length, dry biomass, total chlorophyll, carotenoid, peroxidase (POD), and phenylalanine ammonia lyase (PAL) activity over pathogens-challenged tomatoes. This study is anticipated to serve as an essential indication for synthesis of efficient nano-control agents, which would aid in the management of fatal phyto-pathogens causing significant losses to agricultural productivity. Overall, our findings imply that Ag@*Cf*L-NPs as nano-pesticides might be used in green agriculture to manage the diseases and promote plant health in a sustainable way.

## Introduction

Nanotechnology has evolved as a significant and exciting area of research, with distinctive properties and broad applications in fields such as agriculture, food, and medicine (Erci et al., 2018). Metal and metal oxide (MO) nanoparticles (NPs) have been thoroughly researched using science and technology due to their outstanding properties such as the surface area to volume ratio, high diffusion in solution, and so on (Das Purkayastha and Manhar, 2016). As a result of this, Metal and metal oxide nanoparticles have improved the antibacterial capabilities (Ingle et al., 2020). Silver nanoparticles (Ag-NPs) are gaining lot of attention in the research community because of their wide range of applications in microbiology, chemistry, food technology, cell biology, pharmacology, parasitology and so on (Bondarenko et al., 2013; Nour et al., 2019). The physical and chemical properties of silver nanoparticles are determined by their morphology (Bondarenko et al., 2013). Although higher concentration of silver (Ag) are harmful, however, several investigations have shown that lower concentrations of AgNO_3_ have superior chemical stability, catalytic activity, biocompatibility, with inherent therapeutic potential (Fahimirad et al., 2019). The Ag-NPs have been shown to exhibit the anticancer and antibacterial activity (Haggag et al., 2019). Several approaches, including the sol-gel method, hydrothermal method, chemical vapor deposition, thermal decomposition, microwave-assisted combustion method and others, have been used to synthesize Ag-NPs (Wu et al., 2012). However, while some of these physiochemical approaches are durable and theoretically viable; their large-scale applicability is severely limited due to the use of toxic chemicals, high cost, high energy and time consuming, and difficulties in waste purification. Furthermore, by-products of chemical processes are hazardous to the environment. As a result, there is a rising demand to adopt cost-effective, ecologically safe, and green synthesis techniques that use non-toxic chemicals in nano silver manufacturing methodology. Green synthesis of Ag-NPs employing various microbes, plants, and algae, on the other hand, is a natural, biocompatible, and ecologically friendly method (Aziz et al., 2015). Plant components such as leaves, roots, flowers, fruits, rhizomes, etc., have been effectively used in the synthesis of Ag-NPs (Ahmad et al., 2017; Sumitha et al., 2018). Secondary metabolites found in plants such as alkaloids, amines, proteins, polysaccharides, polyphenols, flavonoids, tannins, terpenoids, ketones, and aldehydes that act as reducing, stabilizing, and capping agents in the conversion of metal ions to metal nanoparticles, resulting in the formation of desirable nanoparticles with predefined properties (Rajan et al., 2015).

Much research has been carried out on the green synthesis of Ag-NPs using plant leaves, however, biosynthesis of Ag-NPs utilizing flower extract demonstrating potential anticancer and antibacterial action has not been extensively investigated. In this context, *Cassia fistula* (L.) is also known as Indian labrum and Golden Shower in English is thought to be an Indian native plant. In Indian literature, it is commonly utilized in the treatment of both common and acute ailments. This herb is also used in the treatment of hematemesis, pruritus, leukoderma, and diabetes (Alam et al., 1990). The indigenous people of Similia Biosphere Reserve (SBR) use this herb for a variety of ethnomedicinal purposes. The floral extract has antibacterial, antifungal (Duraipandiyan and Ignacimuthu, 2007), antioxidant (Bhalodia et al., 2011), and anti-diabetic properties (Manonmani et al., 2005).

Several biotic (pest and pathogens) and abiotic (temperature, drought, and salinity stress) stresses have detrimental impact on crop yield under changing climatic scenario (Malhi et al., 2021). Since last few decades, agriculture faces a variety of challenges, such as indiscriminate application of synthetic fertilizers, pesticides and other chemical inputs. Due to the inefficient delivery system, there is significant loss of active ingredients (10–75 percent) took place through biodegradation, leaching, and immobilization which ultimately affect the core outcomes (Servin et al., 2015). In addition, disease resistance, toxicity to non-target microbes, and human health risks are also serious problems (Deshpande et al., 2017). To reduce the disease severity with increase in crop production and productivity, an integrated strategy is needed to enhance resistance to biotic and abiotic stresses in plants with maximizing resource use efficiency (He et al., 2016). As a result, there has been a surge in interest in using nanomaterial to create nano-formulations that can replace traditional inputs with higher efficacy, lower input cost, and lower environmental damage (Toksha et al., 2021).

Tomatoes (*Solanum lycopersicum* L.) are the second most valuable crop in the world, yet they are susceptible to several diseases caused by bacteria, fungi, and nematodes (Singh et al., 2017). Tomato root infections are one of the most significant obstacles to profitable tomato cultivation across the world. Soil-dwelling fungus, *Rhizoctonia solani* causes root diseases in different edible crops, including tomatoes. *Pseudomonas syringae*, soil-borne plant pathogenic bacteria cause bacterial speck disease in tomatoes and other crops (Mohammed et al., 2020). The most damaging and destructive diseases of tomatoes are those produced by root-knot nematodes, *Meloidogyne incognita* (Wubie and Temesgen, 2019). This infection reduces the tomato production by 38–56% in India. Root-knot nematodes are the most yield-limiting plant-parasitic nematodes (RKNs; *Meloidogyne* spp.). They are found throughout the world (Jones et al., 2013).

Till date chemical pesticides are used to manage the bacterial, fungal, and nematode infections, however, most of them have detrimental effects on human, animal and soil health. In this perception, the use of biosynthesized Ag-NPs in agriculture is gaining popularity due to their antioxidant and broad range of antibacterial action, as well as their eco-friendly, biocompatible, and cost-effective nature (Danish et al., 2021a). It is thus of great interest to investigate the inhibitory impact of manufactured Ag-NPs against important plant pathogens, which may be employed as a cost-effective, ecologically friendly, and safe technique in the field of nanotechnology. In pot trials, the application of increasing concentration of CuO (copper oxide)nanoparticles dramatically decreased the development of wilt symptoms in *Solanum melongena* (L.) infected by *Verticillium dahliae* and increased the biomass by 64% and Cu content by 32% in roots compared to untreated control plants (Elmer and White, 2016). Looking the importane of diseases, the aim of the present study was to investigate the potential of long-lasting Ag@*Cf*L-NPs (*C. fistula* extract to manufacture a biodegradable, natural product nano-silver with no danger of chemical toxicity) to inhibit the diseases-causing phytopathogenic microbes; *Rhizoctonia solani*, *P. syringae*, and *M. incognita* in tomato by enhancing the growth, photosynthetic attributes, lycopene content and defense responses. The Ag@*Cf*L-NPs were phytogenically synthesized and utilized to assess the anti-microbial activities in both *in vitro* and *in vivo* conditions. The anti-biofilm and anti-exopolysaccharides characteristics, as well as NPs-induced alterations in the morphology and cellular permeability in bacterial pathogens were also assessed. Biological attributes (plant length and dry matter), photosynthetic characteristics and enzymatic activities in tomatoes inoculated with plant pathogens and treated with Ag@*Cf*L-NPs were also assessed.

## Materials and methods

### Collection and preparation of leaf extract and chemical used in the study

Fresh *Cassia fistula* (L.) leaves were obtained from the Aligarh Muslim University (AMU) campus, India (27°52’N latitude, 78°51’E longitude, and 187.45 m altitude). Taxonomists from the Department of Botany, AMU examined and confirmed the plant species. To eliminate the dust particles, the fresh leaves were properly cleaned with sterilized double deionized water (ddH_2_O). The leaves were then carefully macerated using pestle and mortar in sterile ddH_2_O to generate a 10% (w/v) leaves broth, which was then heated for a few minutes. After cooling, the resultant extract was filtered through a muslin cloth followed by Whatman No. 1 filter paper before being stored at 4°C. Silver nitrate (AgNO_3_; Sisco Research Laboratories: SRL, Pvt. Ltd., India) was procured from Sigma-Aldrich, United State.

### Synthesis of silver nanoparticles

The silver nanoparticles used in the present study were prepared by using the bio-reduction method of an aqueous solution of AgNO_3_. The bio-reduction method is among the most reliable and extensively investigated methods for the synthesis of Ag-NPs (Jalal et al., 2016; Qais et al., 2020). Briefly, for the preparation of silver nanoparticles, 25 ml extract of *C. fistula* leaf extract was mixed in aqueous solution of AgNO_3_ (100 ml, 1.0 × 10^–3^ mol dm^–3^). The mixture was incubated (allowed to react) for 12 h at 25°Ñ with continuous stirring using a magnetic stirrer. The completion of reduction of AgNO_3_ by the plant extract was indicated by the change in color of the mixture from light green to dark brown. For obtaining silver nanoparticles, the mixture was centrifuged at 15,000 rpm for 20 min to remove any unreacted AgNO_3_ and plant extract. The nanoparticles obtained were washed thrice with double distilled water followed by washing with ethanol. Finally, the synthesized nanoparticles were dried under a vacuum.

### Characterization of silver nanoparticles

The biosynthesized nanoparticles were characterized to confirm their successful synthesis using a UV-visible spectrophotometer (GENESYS 10S UV/VIS, Thermo Fisher Scientific, United States), Scanning electron microscope (SEM; JSM-5600LV, JEOL, Japan), Transmission electron microscope (JEM-2100, JEOL, Japan), X-ray diffractometer (Miniflex II, Rigaku, Japan) equipped with CuKα radiation source (λ = 1.5406 nm), Fourier transform infrared spectrometer (Nicolet iS50, Thermo Fisher Scientific, United States), and Energy-dispersive X-ray spectrometer (JED-2300, JEOL, Japan) following the standard protocols.

### Assessment of anti-phytopathogenic properties of *Cassia fistula* leaf synthesized silver nanoparticles (Ag@*Cf*L-NPs)

#### Anti-bacterial activity against *Pseudomonas syringae*

##### Collection of bacterial pathogen and assessment of cell viability

Bacterial pathogens *Pseudomonas syringae* were obtained from Section of Plant Pathology, Department of Botany, AMU, Aligarh, India. Bacterial strains was purified by re-culturing on Pseudomonas isolation agar (PIA; HiMedia, Mumbai, India) medium. To determine the potentiality of phytogenically synthesized Ag@*Cf*L-NPs, the vitality of the bacterial strain was determined by growing them in the presence of Ag@*Cf*L-NPs (0–400 μg ml^–1^) (under *in vitro* conditions) (refer to Supplementary information section 1.1).

##### Effect of Ag@*Cf*L-NPs on surface morphological changes and cellular permeability

The interaction studies for observing morphological changes in the cells of *P. syringae* treated with Ag@*Cf*L-NPs were done by SEM according to previously described methods of Ansari et al. (2014), Shahid and Khan (2018) and Shahid et al. (2019) (refer to Supplementary information section 1.2). To assess the effects of synthesized nanohybrid (i.e., Ag@*Cf*L-NPs) on cellular permeability (cell membranes) of the pathogenic isolates, confocal laser scanning microscopy (CLSM) was done (Shahid and Khan, 2018; Khan et al., 2020; Shahid et al., 2021a; Syed et al., 2021) (refer to Supplementary information section 1.3).

##### Effect of Ag@*Cf*L-NPs on biofilm formation

The effect of phytogenically synthesized Ag@*Cf*L-NPs on the biofilm development of *P. syringae* was investigated in a 96-well titer plates (Shahid et al., 2021b). In brief, wells were filled with 200 μl of Luria Bertani (LB; HiMedia, Mumbai, India) broth inoculated with bacterial culture (100 μl) and 0–400 μg Ag@*Cf*L-NPs ml^–1^. The bacterial cultures without Ag@*Cf*L-NPs were served as a control. The optical density (O.D) was measured using a scanning microplate spectrophotometer at 570 nm and recorded the biofilm development.

##### Determination of exopolysaccharide in the presence of Ag@*Cf*L-NPs

For the assessment of EPS production, bacterial cells of *P. syringae* were cultured in a liquid culture medium treated with different doses of Ag@*Cf*L-NPs to test the influence of Ag@*Cf*L-NPs on extrapolymeric substances (EPS) produced by bacterial isolates (Ku et al., 2015) (refer to Supplementary information section 1.4).

#### Antifungal activity of Ag@*Cf*L-NPs

Fungal pathogens such as *Fusarium oxysporum*, *Rhizoctonia solani*, and *Sarocladium* sp. were procured from Section of Plant Pathology, Department of Botany, AMU, Aligarh, India. All the fungal phytopathogens was purified by re-culturing on Potato Dextrose agar (PDA; HiMedia, Mumbai, India) medium. The *in vitro* experiments were performed on potato dextrose agar (PDA; HiMedia, Mumbai, India) medium containing different concentrations of Ag@*Cf*L-NPs (0, 25, 50, 100, 200, and 400 μg ml^–1^). Briefly, 10 ml of Ag@*Cf*L-NPs was taken from each concentration and mixed in 100 ml potato dextrose agar (PDA) media before plating (Petri plates, 90 × 15 mm). Petri plates containing Ag@*Cf*L-NPs were incubated at room temperature for 48 h. Thereafter, the phyto-fungal pathogens such as *Rhizoctonia Solani*, *Fusarium oxysporum*, and *Sarocladium* sp. were inoculated simultaneously at the center of each Petri plate containing Ag@*Cf*L-NPs (agar plugs of uniform size, 5 mm in diameter) and incubated at 28 ± 2°C for 7 days. The Petri plates without Ag@*Cf*L-NPs served as control. After 7 days of incubation, radial growth of fungal mycelium was recorded. All the treatments were performed in three replicates (*n* = 3). The inhibition of mycelial growth was measured according to the method given by Kaur et al. (2012) and percent inhibition was calculated using the following equation:

$$\%inhibition=\frac{C-T}{C}\times 100$$

Where,

*C* = Radial growth of fungi in control plates

*T* = Radial growth of fungi in treated plates.

#### Assessment of nematicidal activity of Ag@*Cf*L-NPs

##### *In vitro* activity of silver nanoparticles (Ag@*Cf*L-NPs) on hatching and mortality of *Meloidogyne incognita*

For the recovery of root-knot nematodes, infected roots of eggplant (*Solanum melongena* L.) with the root-knot nematode (*M. incognita*) were collected from an eggplant field. Root-knot nematode species *M. incognita* was identified on the basis of the North Carolina differential host test and perennial pattern morphology. The effect of different concentrations (0, 25, 50, 100, 200, and 400 μg ml^–1^) of Ag@*Cf*L-NPs on egg hatching and mortality of *M. incognita* was studied under *in vitro* conditions. Five (05) egg masses of average sized (collected from eggplant roots) were placed for hatching in small sized (60 × 15 mm) Petri plates containing 10 ml Ag@*Cf*L-NPs solution from each concentration using sterilized forceps and incubated at 25 ± 1°C. Five egg masses placed in 10 ml of ddH_2_O were served as control. Each treatment was repeated thrice (*n* = 3). After 48 h of incubation, the total number of hatched larvae and immobile larvae were counted using a nematode counting dish under a stereoscopic microscope. The death of the juveniles was determined by immersing the immobile larvae in water for 1 h and counting the number of dead larvae.

#### Effect of Ag@*Cf*L-NPs on phytopathogen-infected tomato crop

##### Effect of Ag@*Cf*L-NPs on biological attributes of tomato

Efficacy of green-synthesized Ag@*Cf*L-NPs was evaluated in *Solanum lycopersicum* L. (tomato) against root-knot caused by *Meloidogyne incognita*, root rot caused by *Rhizoctonia solani*, and bacterial speck diseases caused by *Pseudomonas syringae*. Tomato seeds were disinfected with four percent sodium hypochlorite (NaOCl; Sisco Research Laboratories: SRL, Pvt. Ltd., India) for 15 min and then washed three times with distilled sterile water. The inoculum of microbial phytopathogens was applied as; *P. syringae* (10^8^ CFU ml^–1^), *R. solani* (50 g mycelial mat), and *M. incognita* (1,000 J_2_) (Kaur et al., 2016). To evaluate the disease suppressive and plant growth-promoting effect of Ag@*Cf*L-NPs, the two concentrations (50 and 100 mg kg^–1^) were used in the tomato experiments (of the two-leaf growth stage). Sterile distilled water (SDW) was used as a negative control. There was eighth different treatments, i.e., (i) Control: water only (negative control), 50 mg Ag@*Cf*L-NPs kg^–1^ only (positive control), 100 mg Ag@*Cf*L-NPs kg^–1^ only (positive control), (ii) Inoculated control: *Meloidogyne incognita* only, *R. solani* only, *P. syringae* only, (iii) *R. solani* + 50 mg Ag@*Cf*L-NPs kg^–1^, (iv) *M. incognita* + 50 mg Ag@*Cf*L-NPs kg^–1^, (v) *P. Syringae* + 50 mg Ag@*Cf*L-NPs kg^–1^ (vi) *M. incognita* 100 mg Ag@*Cf*L-NPs kg^–1^, (vii) *R. solani* + 100 mg Ag@*Cf*L-NPs kg^–1^, and (viii) *P. syringae* + 100 mg Ag@*Cf*L-NPs kg^–1^. At 45 days after inoculation (DAI), the pathogen-infected and Ag@*Cf*L-NPs treated tomatoes were harvested and cleaned the roots by removing the adhering peat. The plant growth parameters (root and shoot length, fresh and dry biomass), chlorophyll content, lycopene content, and antioxidant enzymes were estimated. In the case of nematode inoculated plants, the number of galls per root system were enumerated and used to calculate the root-knot index (RKI) and the percentage (%) of root rot per root system was visually inspected. Furthermore, percent disease incidence was assessed from the fungal infected and Ag@*Cf*L-NPs-treated tomato plants.

##### Chlorophyll pigment and lycopene content estimation

At harvest, from the phytopathogens-inoculated and Ag@*Cf*L-NPs-treated tomato leaf, photosynthetic pigments (total chlorophyll and carotenoid contents) were measured by spectrophotometer (UV-visible spectrophotometer; GENESYS 10S UV/VIS, Thermo Fisher Scientific, United States) following the procedure of MacKinney (1941).

##### Antioxidant enzymatic activities

*Peroxidase* (POD) activity was determined by monitoring the absorbance at 470 nm every 20 s using the spectrophotometer (Ramzan et al., 2021). Determination of superoxide dismutase (SOD) activity was performed according to Giannopolitis and Ries (1977), while catalase (CAT) activity was estimated as per methods described by Danish et al. (2021b). The activity of phenylalanine ammonia-lyase (PAL) was spectrophotometrically (UV-visible spectrophotometer; GENESYS 10S UV/VIS, Thermo Fisher Scientific, United States) determined in the plant treated with pathogens and Ag@*Cf*L-NPs as per the method given by Solekha et al. (2020) (Please refer to the respective Supplementary information sections 2.1 and 2.2 in Supplementary Information).

### Effect of Ag@*Cf*L-NPs on gall formation, root-knot index (RKI), and disease incidence in tomatoes

The *M. incognita* inoculated and Ag@*Cf*L-NPs-treated tomatoes were harvested and the number of galls per plant was visually counted. The size of each gall was measured using a micrometer to determine its maximum length and breadth (in m). With minor modifications, the severity of the disease was measured using the grading scale 0–6 given by Popoola et al. (2015).

### Statistical analysis

*In vitro* and greenhouse studies were carried out in a complete randomized block design (CRBD), and data were analyzed through analysis of variance (ANOVA) at *p* < 0.05 level using the statistical software R. Pearson correlation matrix between variables was performed by XLSTAT.

## Results and discussion

### Characterization of Ag-NPs

#### UV-vis spectrophotometric analysis

**FIGURE 1:**
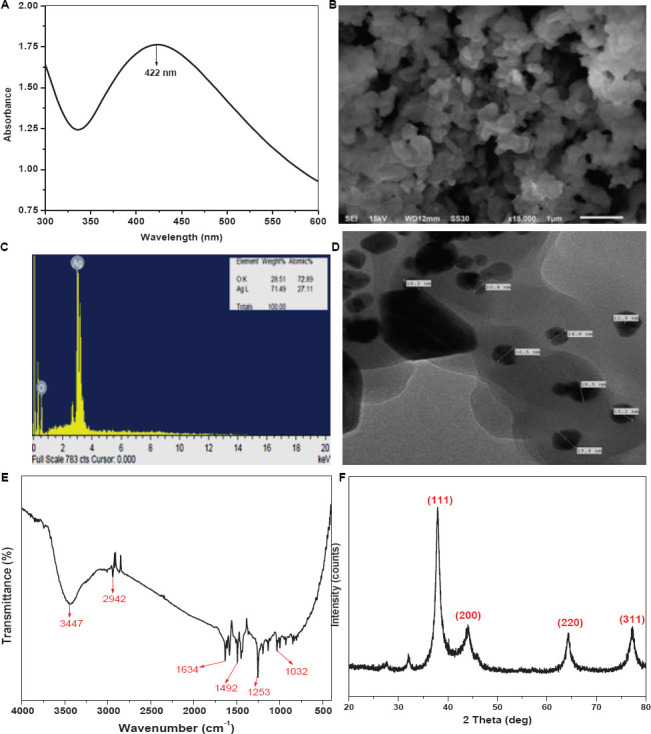
Characterization of *Cassia fistula* leaf synthesized silver nanoparticles (Ag@*Cf*L-NPs): UV–vis spectra of Ag@*Cf*L-NPs **(A)**, scanning electron micrographs (SEM) of Ag@*Cf*L-NPs **(B)**, EDX spectrum showing the histogram of wt% of major elements in Ag@*Cf*L-NPs **(C)**, transmission electron micrographs (TEM) of Ag@*Cf*L-NPs **(D)**, FTIR spectrum of Ag@*Cf*L-NPs **(E)** and XRD pattern of powdered Ag@*Cf*L-NPs **(F)**.

UV-visible spectroscopy is one of the most reliable methods for the characterization of silver nanoparticles. In Ag-NPs, the conduction band lies very close to the valence band, so the valence band electrons, excited by the photon of the incident light, can easily jump into the conduction band and move freely. The freely moving electrons are responsible for the Surface Plasmon Resonance (SPR), which occurs because of the collective oscillation of electrons in response to the incident light (Zhang et al., 2016; Rezazadeh et al., 2020). A broad absorbance peak was observed at 422 nm for the colloidal solution of Ag@*Cf*L-NPs, which is associated with the SPR band of Ag@*Cf*L-NPs and confirms the stability of NPs (Figure 1A).

#### Transmission electron microscopy (TEM) and scanning electron microscopy (SEM)

**FIGURE 2:**
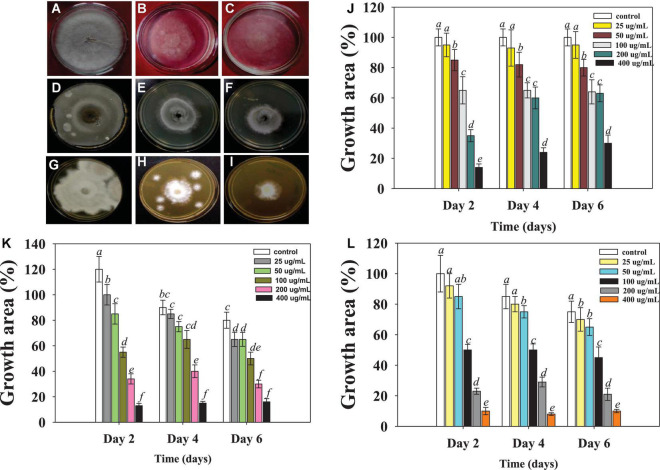
Macroscopic examination depicting the Ag@*Cf*L-NPs-induced inhibition in mycelia growth of phytopathogenic fungi; *Rhizoctonia solani*: control **(A)**, treated with 50 **(B)**, 100 μg ml^–1^ Ag@*Cf*L-NPs **(C)**. *Sarocladium* sp.: control **(D)**, treated with 50 **(E)**, 100 μg Ag@*Cf*L-NPs ml^–1^ **(F)**, *Fusarium oxysporum*: control **(G)**, treated with 50 **(H)**, 100 μg ml^–1^ Ag@*Cf*L-NPs **(I)**. In this figures, **(J–L)** represents the bar diagrams showing the numerical data in terms of percentage of growth area as compared to mycelia growth of untreated control done in triplicates (*n* = 3). Corresponding error bars represents standard deviation (S.D) of three replicates (S.D, *n* = 3).

To study the surface features of the synthesized NPs, the Ag@*Cf*L-NPs, were characterized using SEM and TEM. The SEM image (Figures 1B,C) of Ag@*Cf*L-NPs clearly shows that the shape of the NPs was mostly spherical and some of the Ag@*Cf*L-NPs were oval as well. The SEM images demonstrate the bio-molecule coating of the synthesized Ag@*Cf*L-NPs. This layer confirms the significance of plant extract metabolites in the synthesis and stabilization of Ag@*Cf*L-NPs. These findings are consistent with those of the observation of Oves et al. (2018). Further, to study the topographical and crystallographic characteristics of the NPs TEM imaging was performed. The TEM image of synthesized Ag@*Cf*L-NPs (Figure 2) depicts that the NPs vary in size and range between 10 and 20 nm in diameter. Also, the shape of Ag@*Cf*L-NPs observed in the TEM micrograph is following the SEM image, i.e., spherical. This finding was in lined with the results of Danish et al. (2021a).

#### Energy dispersive X-ray (EDX) analysis

For elemental analysis, the prepared Ag@*Cf*L-NPs were characterized using EDX (Figure 1). The result of EDX analysis for synthesized Ag NPs depicts a very high content of Ag (71%) with some O (28%) that may be absorbed on the surface of synthesized Ag@*Cf*L-NPs. This confirmed the existence of the silver element in the synthesized Ag@*Cf*L-NPs.

#### Fourier transform infrared spectroscopy (FTIR)

The characterization of complicated and particular materials using Fourier Transform Infrared Spectrometry (FTIR) is a difficult assignment for chemists to complete. To study the nature of functional groups present in the Ag@*Cf*L-NPs, the synthesized NPs were characterized using FTIR (Figure 1E). The peak observed at 3,447 cm^–1^ is due to the O–H stretching vibrations of the phenol group (Zia et al., 2016). The absorbance peak at 2,942 cm^–1^ is attributed to the C–H stretching vibrations (Manikprabhu and Lingappa, 2013). The stretching vibrations of C = C are observed at 1,634 cm^–1^ (Jyoti et al., 2016). The peaks obtained at 1,492 cm^–1^ and 1,253 cm^–1^ are assigned to C–C stretch and C–N stretch, respectively (Devaraj et al., 2013). And the peak observed at 1,032 cm^–1^ corresponds to the stretching vibrations of the C–OH group (Singh et al., 2014).

#### X-ray diffraction (XRD)

The analytical technique of X-ray diffraction (XRD) is based on the diffraction of X-rays by matter, particularly crystalline materials. The Ag@*Cf*L-NPs were characterized using XRD to confirm the crystalline nature of the NPs. Four intense peaks were observed in the XRD pattern of Ag@*Cf*L-NPs (Figure 1F) at diffraction (2θ) angles of 37.92°, 44.04°, 64.38°, and 77.32°, which corresponds to the planes 111, 200, 220, and 311, respectively (Remya et al., 2015; Sigamoney et al., 2016), indicating the crystalline nature of Ag-NPs (Abbasi et al., 2020). The average crystallite size, calculated with a peak at 2θ = 37.92° using the Debye-Scherrer formula was 14.63 nm.

### The anti-phytopathogenic potential of Ag@*Cf*L-NPs

#### Effect of Ag@*Cf*L-NPs on phytopathogenic bacterium *Pseudomonas syringae*

The growth of bacterial pathogen *Pseudomonas syringae* was hindered by the increasing concentrations Ag@*Cf*L-NPs, which showed a strong antibacterial potential. This study is intriguing from an agronomic standpoint because *P. syringae*, like other bacterial diseases, causes major crop losses in various agriculturally important crops including tomatoes. For example, bacterial speck disease caused by *P. syringae* damages a variety of agricultural products, including tomatoes and tobacco. As a result, breeding of resistant cultivars, cultural practices, and crop rotation are some of the potential traditional measures used to control the negative consequences of *Pseudomonas syringae*.

#### Growth and viability

**FIGURE 3:**
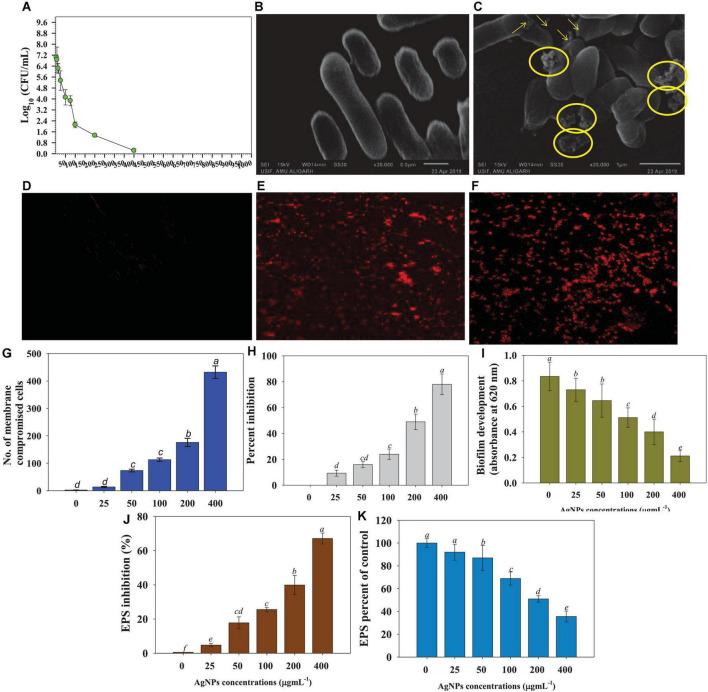
Anti-bacterial properties of increasing concentrations of *Cassia fistula* leaf synthesized silver nanoparticles (Ag@*Cf*L-NPs) against soil bacterial pathogen *Pseudomonas syringae*; effect of 0–1,000 μg Ag@*Cf*L-NPs ml^–1^ on CFU count **(A)**, morphological changes: control cells of *P*. *syringae* **(B)** treated with 100 μg Ag@*Cf*L-NPs ml^–1^ **(C)**, NP-induced cellular permeability: CLSM images of untreated bacterial cells showing no bacterial uptake of propidium iodide **(D)**, treated with 100 μg Ag@*Cf*L-NPs ml^–1^ **(E)** and 200 μg Ag@*Cf*L-NPs ml^–1^ showing the red-colored rod-shaped cells **(F)**, number of membrane compromised cells **(G)**, percent inhibition in bacterial biofilm formation **(H)**, biofilm development in the presence of increasing doses of Ag@*Cf*L-NPs **(I)**, percent inhibition in bacterial EPS **(J)** and EPS percent of control **(K)**. In this figure, bar diagrams represent the mean values of three replicates (*n* = 3). Corresponding error bars represents standard deviation (S.D) of three replicates (S.D, *n* = 3).

The effectiveness of such techniques, on the other hand, has been proven to be highly variable. As a result, the antibacterial activity of Ag@*Cf*L-NPs as demonstrated in this study may be critical in controlling the bacterial phytopathogens. The high surface energy and mobility of nanoparticles are influenced by strong contacts between Ag-NPs and the cell wall. The findings of this investigation revealed that Ag@*Cf*L-NPs at various concentrations displayed potent antibacterial action against *Pseudomonas syringae*, with the inhibitory impact increasing as the concentration was raised. The diameter of the inhibitory zones was 12.2 mm in the presence of 400 μg Ag@*Cf*L-NPs ml^–1^. Previous research has shown that green synthesized Ag-NPs can reduce a variety of bacterial plant diseases, including *Pseudomonas syringae* pv. tomato (Marpu et al., 2017), *Clavibacter michiganensis* subspecies *michiganensis* (Rivas-Cáceres et al., 2018), and *Ralstonia solanacearum* (Chen et al., 2016), which are all in accordance with our findings. Furthermore, the viability of bacterial cells was significantly reduced as the concentration of Ag@*Cf*L-NPs was increased (Figure 3A).

#### Ag@*Cf*L-NPs-induced morphological changes in bacteria

Under a scanning electron microscope (SEM), morpho-structural alterations caused by synthesized Ag@*Cf*L-NPs in *P. syringae* were studied. Bacterial cells with no treatment (control) have smooth cell surfaces and shapes (Figure 3B). However, as shown in pictures with yellow arrows and circles, Ag@*Cf*L-NPs-treated cells showed a clear damage to the outer surface, shrinkage, and broken membranes (Figure 3C). The peptidoglycan layer of two different bacterial cells differs, resulting in this morphological discrepancy. However, the precise method by which Ag@*Cf*L-NPs inhibit or kill the microorganisms is still unknown. However, other processes cause Ag-NPs to become toxic and eventually contribute to the death of bacterial cells, such as decoupling oxidation, free radical formation, interferences with the respiratory chain of cyt c, interference with the transport chain’s components of the microbial electron chain, phosphorous and sulfur compounds, and DNA interactions (Lapresta-Fernández et al., 2012).

#### Membrane permeability of plant pathogenic bacteria

The qualitative analyses of cell permeability in *P. syringae* were performed using the fluorescently labeled probe propidium iodide (PI). Due to the staining of PI with bacterial DNA, the 400 μg Ag@*Cf*L-NPs ml^–1^ -treated cells looked red against the black background, followed by excitation at 532 nm (Figures 3D–F). This is because PI binds to DNA molecules in the membrane-damaged cells (Stiefel et al., 2015). Furthermore, the number of dead cells in Ag@*Cf*L-NPs-treated membrane-impaired bacterial cells was quantified, and it was observed that the number of dead cells increased in a dose-dependent way (Figure 3G). The antibacterial properties of nanoparticles are dependent on particle size, stability, and concentration in the growth medium (Azam et al., 2012). The NPs have a stronger reactivity with pathogens because the outer cellular membrane of the bacterial strains has nano-scale pores. As a result of the ROS, the bacterial cell membrane is damaged, allowing the cells to absorb PI. Furthermore, the antibacterial effect of Ag@*Cf*L-NPs is principally due to the formation of reactive oxygen species (ROS) such as OH^–^, H_2_O_2_, and O_2_.

#### Biofilm formation and exopolysaccharide production

The results of this investigation showed that at all doses of synthesized Ag@*Cf*L-NPs in this study greatly decreased the biofilm formation ability of *P. syringae*. The Ag@*Cf*L-NPs at concentrations of 200 and 400 μg ml^–1^ caused a greater inhibition in optical density (OD) values, with decreases of 49 and 78%, respectively, over control (Figures 3H,I). Biofilms are bacterial aggregation that may survive in harsh environments and are resistant to the immune system of the host as well as certain chemotherapeutic treatments. Biofilm development protects the bacteria from external stimuli and keeps them alive, whereas swimming motility permits the plant pathogenic bacteria to easily infiltrate and colonize the host plants (Denny, 2007). As a result, the antibacterial action of Ag@*Cf*L-NP scan be ascribed in part to their prevention of bacterial biofilm formation. The existence of reactive oxygen species (ROS) as a primary inhibitor of biofilm formation was further clarified in the study. As a result, the enhanced generation of extracellular ROS might explain this investigation. Like our study, bio-fabricated AgNPs showed a strong anti-bacterial efficacy and reduced the biofilm formation activity of soil-borne pathogenic bacterium *Xanthomonas oryzae* that causes leaf blight diseases of rice (Mishra et al., 2020). Furthermore, ZnO nanoparticles demonstrated a significant reduction of bacterial growth and biofilm formation against *P. aeruginosa*, as previously reported by Dwivedi et al. (2014). They also identified that reactive oxygen species (ROS) as a key mechanism for ZnO-NP action. With increasing concentrations, Ag@*Cf*L-NPs, the formation of EPS by plant pathogenic bacteria was significantly (*p* ≤ 0.05) reduced. The higher rates of synthesized nanomaterial had the maximum toxic effect on EPS. For example, at 400 μg ml^–1^, Ag@*Cf*L-NPs reduced the EPS formation by 76% over untreated control (Figures 3J,K).

#### Antifungal potential of Ag@*Cf*L-NPs against major fungal pathogens

The antifungal potential of phytogenically synthesized Ag@*Cf*L-NPs was evaluated against major fungal pathogens (*Rhizoctonia solani*, *Sarocladium* sp. and *Fusarium oxysporum*) causing yield losses in edible crops. All the concentrations of synthesized Ag@*Cf*L-NPs had significant (*p* ≤ 0.05) inhibitory effect on the growth of tested fungal pathogens (Supplementary Table 1). The Ag@*Cf*L-NPs-induced dose-dependent inhibition in mycelial growth of *Rhizoctonia* (Figures 2A–C), *Sarocladium* (Figures 2D–F), and *Fusarium* (Figures 2G–I) were observed which, differed considerably with time. However, no consistent link was found between fungal growth suppression and incubation times. When cultivated on potato dextrose agar (PDA; HiMedia, Mumbai, India) plates supplemented with 400 μg Ag@*Cf*L-NPs ml^–1^, the growth of *R. solani* was decreased by 65%, 75%, 78% after 2nd, 4th, and 6th days of incubation, respectively (Figure 2J). Likewise, at 400 μg Ag@*Cf*L-NPs ml^–1^, the growth of *F. oxysporum* was inhibited by 24, 56, and 73% at 2nd, 4th, and 6th days of incubation, respectively (Figure 2K). It was clear from this study that each concentration of Ag@*Cf*L-NPs had a harmful effect on fungal growth, while at higher concentration (400 μg ml^–1^), the growth was completely stopped in the tested fungal phytopathogens. Other researchers have also reported that NPs have antifungal properties. ZnO-NPs, for example, hindered the growth of *Botrytis cinerea* and *Penicillium expansum* by deforming the structure of fungal hyphae and stopping the conidial germination and development (He et al., 2011). Likewise, Elgorban et al. (2016) confirmed that varying concentrations of Ag-NPs have antifungal properties against *R. solani* and a strong inhibition was noticed on CDA at different concentrations. The phyto-synthesized Ag-NPs completely inhibited the growth of *R. solani* by increasing lipid peroxidation (ROS generation). It may be the primary cause of toxicity of Ag-NPs in the fungal cell (Kanaujiya et al., 2020). In addition, results showed that cell surfaces of fungal hyphae was distorted. Aside from that, Ag-NPs interfere with the membrane transport processes, such as ion efflux (Rudakiya and Pawar, 2017). The deformed membranes may allow silver ions generated by Ag-NPs to accumulate in the nutritional medium, altering cellular respiration and metabolic processes (Nisar et al., 2019). Another process that negatively affects are lipids, proteins, and nucleic acids due to the generation of intracellular ROS.

#### Effects of Ag@*Cf*L-NPs on hatching and mortality of root-knot nematode *Meloidogyne incognita*

**FIGURE 4:**
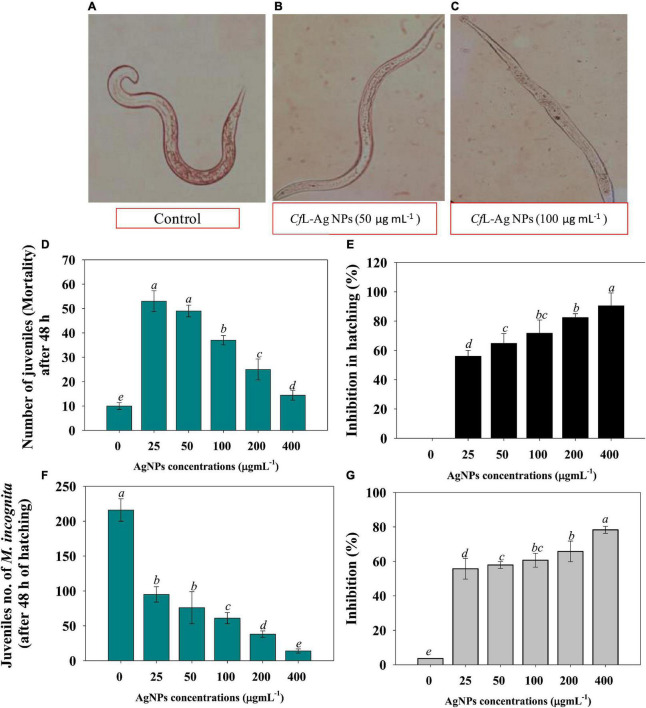
**(A–C)** Represent the microscopic examination of root-knot nematode *Meloidogyne incognita* treated with 0 (control), 50 and 100 μg Ag@*Cf*L-NPs ml^–1^, respectively. **(D,E)** Shows the effect of Ag@*Cf*L-NP son number of juveniles of *M. incognita* after 48 h of hatching and percent inhibition in hatching, respectively. **(F,G)** Depicts the impact of green-synthesized Ag@*Cf*L-NP son number of juveniles (mortality) after 48 h and inhibition percentage, respectively. In this figure, bar diagrams represents the mean values of three replicates (*n* = 3). Corresponding error bars represents standard deviation (S.D) of three replicates (S.D, *n* = 3).

To assess the nematicidal properties of Ag@*Cf*L-NPs, different concentrations of synthesized Ag-NPs solution (0–400 μg ml^–1^) were tested on the hatching and mortality of root-knot nematode, *M. incognita*. All the concentrations of Ag-NPs had a significant (*p* ≤ 0.05) inhibitory effect on the hatching and mortality of *M. incognita* (Supplementary Tables 2, 3). For instance, a reduction of 56, 64, 71, and 82% in egg hatching of *M. incognita* was recorded when treated with 25, 50, 75, and 100 μg Ag@*Cf*L-NPs ml^–1^, respectively, after 48 h as compared to untreated control (Figures 4D,E). The adverse effect encountered might be due to the toxicity of green synthesized Ag@*Cf*L-NPs that inhibited the hatching of *M. incognita*. Similarly, increasing doses of Ag-NPs reduced the egg hatching of *M. javanica* as previously reported by Hamed et al. (2019). The inhibition effect of Ag-NPs was correlated with the physical properties (size, shape, and homogeneity), which may play a crucial role in the cell wall penetration in the eggs followed by dysfunctioning in cells (Jagtap and Bapat, 2013). However, larval mortality of *M. incognita* (2nd stage) was 4.62% in double-distilled water after 48 h. The juvenile mortality of *M. incognita* in Ag-NPs (at 25, 50, 75, and 100 mg/L) was observed to 55.78, 57.89, 60.65, and 65.78%, respectively, after 48 h of the exposure period (Figures 4F,G). Microscopic observation revealed that after treatment of 100 and 200 μg Ag@*Cf*L-NPs ml^–1^ to 2nd stage juveniles of *M. incognita* used a remarkable deformation in nematode structure (Figures 4A–C). Baronia et al. (2020) observed that direct exposure of Ag-NPs in a solution of water had strong nematicidal potential at low concentration against mortality of *M. graminicola* (J2) irreversibly within 12 h. In a similar study, exposure to Ag-NPs showed cell wall degradation under *in vitro* conditions and ultimately caused mortality of *M. incognita* at different exposure times (Hassan et al., 2016).

### Assessing the disease suppressive and growth promotion potential of Ag@*Cf*L-NPs in tomato crop

The Ag@*Cf*L-NPs were drenched in the soil to assess the effectiveness of Ag@*Cf*L-NPs in controlling soil-borne plant pathogenic microbes and plant growth-promoting effects in tomatoes in terms of growth factors and disease development. In the present study, tomatoes were chosen because they are most susceptible to phytopathogens (bacteria, fungi, and nematodes). Fungal pathogens cause severe diseases in different edible crops, including tomatoes. *Pseudomonas syringae*, soil-borne plant pathogenic bacteria cause bacterial speck disease in tomatoes and other crops. The most damaging and destructive diseases of tomatoes are those produced by root-knot nematodes *Meloidogyne incognita*.

#### Effect of Ag@*Cf*L-NPs on biological attributes of tomatoes

**FIGURE 5:**
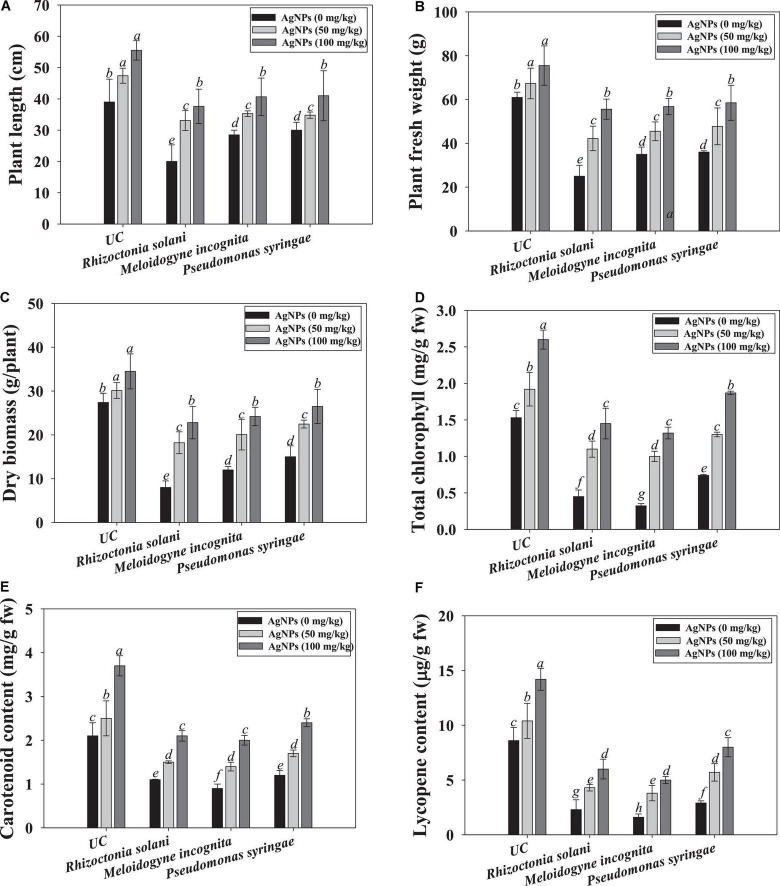
Effect of *Cassia fistula* leaf synthesized silver nanoparticles (Ag@*Cf*L-NPs) on biological and physiological parameters of tomato crop cultivated in pot-soils infected with phytopathogens; *R. solani* (fungi), *M. incognita* (root-knot nematodes) and *P. syringae* (bacteria): plant length **(A)**, fresh weight **(B)**, dry biomass **(C)**, total chlorophyll **(D)**, carotenoid content **(E)** and lycopene content **(F)**. In this figure, bar diagrams represents the mean values of three replicates (*n* = 3). Corresponding error bars represents standard deviation (S.D) of three replicates (S.D, *n* = 3).

Under phytopathogen-challenged conditions, the biological parameters of tomatoes were greatly reduced. For instance, dry biomass was reduced by 45, 56, and 67% when tomatoes were infected with *R. solani*, *P. syringae*, and *M. incognita*, respectively. However, findings showed that phytogenically synthesized Ag@*Cf*L-NPs greatly aided the tomato seedling development when compared to the pathogen-inoculated plants. The effects of Ag@*Cf*L-NPs on biological attributes *viz*., growth, length, and dry biomass were varied significantly. For example, at 100 mg Ag@*Cf*L-NPs kg^–1^, plant length, and dry biomass of tomatoes were greatly increased by 66 and 58%, respectively, over NPs-untreated and pathogen infected plants (Figures 5A–C). The green synthesized Ag@*Cf*L-NPs reduced the growth of phytopathogens and improved the growth and biometric parameters of tomatoes cultivated even in pathogen-challenged plants. Previous research has found that green synthesized NPs including Ag-NPs have a favorable influence on plant development, which is consistent with the findings of this study. Plant resilience to stress may be improved by NPs with antioxidant enzyme capabilities, which improve the capacity of plants to scavenge ROS and hence reduce the yield losses (Zhang et al., 2018). In hydroponics studies, Liang et al. (2018) found that the Cos–La nanoparticles greatly improved the development of rice plant by increasing root length and fresh weight and ultimately improve the defense response. Similarly, varied concentrations of Ag-NPs encouraged the growth of tulip plants by raising the leaf greenness index, stomatal conductance, length and fresh weight of roots (Byczyńska et al., 2019).

#### Photosynthetic pigments and lycopene content

The ability of Ag@*Cf*L-NPs to maintain chlorophyll integrity suggests that following their application, the structure’s defense mechanisms and photosynthetic pigments are activated in the tomatoes. The photosynthetic properties of tomato leaf were decreased when plants were grown in soil inoculated with phytopathogenic microbes. However, Ag@*Cf*L-NPs had a considerable and positive impact on the concentration of chlorophyll and carotenoids in tomato leaves. The 100 mg Ag@*Cf*L-NPs kg^–1^ treatment had the highest results in terms of chlorophyll and carotenoid concentration, outperforming control (Figures 5D,E).

Lycopene, a carotenoid found naturally in tomatoes, is also responsible for the production of antioxidant molecules. As a result, lycopene-rich meals are extremely healthy. Lycopene content was dramatically reduced when soil pathogenic microbes were inoculated to tomato crops. When Ag@*Cf*L-NPs were used instead of pathogen-infected treatment, the lycopene content of tomato fruit rose significantly. For instance, at 100 mg Ag@*Cf*L-NPs kg^–1^, lycopene content in tomato fruits was increased by 43, 52, and 38% when applied to soils inoculated with *R. solani*, *P. syringae*, and *M. incognita*, respectively, over NP-untreated but inoculated plants (Figure 5F). Similar to our study, *Cassis fistula* leaf synthesized Cu oxide nanoparticles (CuO-CFNPs), improved the lycopene content in tomato fruits raised in soil infected with *Fusarium oxysporum* by inhibiting fungal growth (Ashraf et al., 2021).

#### Antioxidant enzymatic activity of Ag@*Cf*L-NPs-treated tomato plants

**FIGURE 6:**
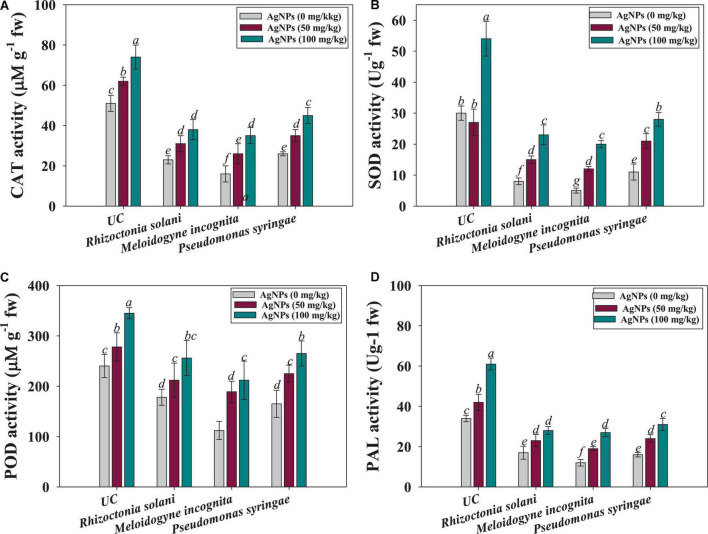
Effect of Ag@*Cf*L-NP son antioxidant enzymatic activities in leaf tissues of tomato crop cultivated in pot-soils infected with phytopathogens; *M. incognita* (root-knot nematodes) *P. syringae* (bacteria) and *R. solani* (fungi): catalase; CAT **(A)**, superoxide dismutase; SOD **(B)**, peroxidase; POD **(C)** and phenylalanine ammonia lyase; PAL **(D)**. In this figure, bar diagrams represents the mean values of three replicates (*n* = 3). Corresponding error bars represents standard deviation (S.D) of three replicates (S.D, *n* = 3).

In this work, the synthesized silver nanoparticles (Ag@*Cf*L-NPs) generated a high antioxidant response in tomatoes against pathogen such as *P. syringae*, *R. solani*, and *M. incognita* by lowering their population in the soils and reducing the severity of diseases. The application of Ag@*Cf*L-NPs had a considerable impact on antioxidant enzymatic activities such as superoxide dismutase (SOD), peroxidase (POD), catalase (CAT), and phenylalanine ammonia lyase (PAL) accumulated in root tissues of tomatoes cultivated in phytopathogens-challenged soil system. The treatment of 100 mg Ag@*Cf*L-NPs kg^–1^ was constant, showing that all enzymes had their maximal activity. The enzyme peroxidase (POD) is accountable for controlling the metabolic activity, chlorophyll formation, respiration, substratum oxidation, growth development, and infections in plants (Danish et al., 2021b). POD is known to be involved in lignin production; they help plants defend themselves against infections by strengthening plant cell walls and conveying signals to other cells that are unaffected (Walters, 2011). Superoxide dismutase (SOD) could catalyze the conversion of O_2_ to hydrogen peroxide (H_2_O_2_), providing an initial amount of protection against ROS, but antioxidant enzymes like POD, CAT, and APX were able to limit H_2_O_2_ build-up to a minimum, keeping plants safe from oxidative stress (Abedi and Pakniyat, 2010). For instance, CAT activity in Ag@*Cf*L-NPs-treated roots, the same findings were reported; treatment with 100 mg Ag@*Cf*L-NPs kg^–1^ was shown to be 61 percent better than control (Figure 6A). Additionally, among treatments, the maximum improvement of 47 and 60% in SOD (Figure 6B) and POD (Figure 6C) were recorded when 100 mg Ag@*Cf*L-NPs kg^–1^ was applied to *Pseudomonas syringae* challenged tomatoes over nanoparticles untreated and pathogen-infected plants. Likewise, phenylalanine ammonia-lyase (PAL) is an enzyme that triggers the plant defense responses in stressful situations; the creation of phenylpropanoid phytoalexins after fungal infection drives the fast production of PAL (MacDonald and D’Cunha, 2007). The Ag@*Cf*L-NPs at 100 mg kg^–1^ showed the maximum PAL activity in tomato plants, outperforming the control by 68% (Figure 6D). As a result, it is possible that an increase in PAL activity following the administration of Ag@*Cf*L-NPs. Furthermore, the reaction to antioxidant enzymes is dependent on a variety of parameters, including the kind of NP, the quantity of NPs, and the plant species; yet, the current findings are similar to Ashraf et al. (2021). Likewise, Danish et al. (2021b) observed substantial differences in the level of antioxidant enzymes (APX, POD, SOD, and CAT) in ajwain plants raised in soil exogenously inoculated with Ag-NPs and M. *incognita*.

#### Disease attributes (gall formation and disease incidence) in Ag@*Cf*L-NPs-treated tomatoes

**FIGURE 7:**
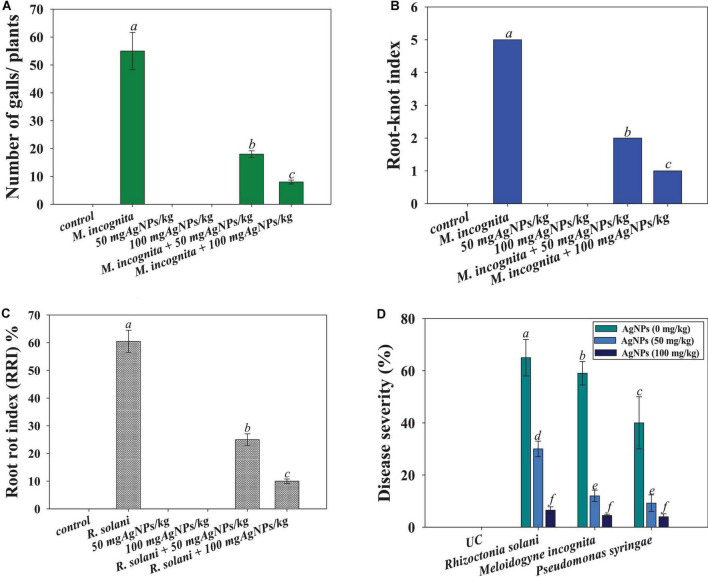
Effect of Ag@*Cf*L-NPs on number of galls caused by *M. incognita* **(A)**, root-knot index **(B)**, root-rot index **(C)** and percent disease severity **(D)** in tomato crop cultivated in pot and infected with phytopathogens; *P. syringae*, *M. incognita*, and *R. Solani*. In this figure, bar diagrams represent the mean values of three replicates (*n* = 3). Corresponding error bars represents standard deviation (SD) of three replicates (S.D, *n* = 3).

Gall development was seen in tomatoes treated with root-knot nematode *M. incognita*. The number of galls was observed to be greatly reduced (76%) when tomatoes were treated with 100 mg Ag@*Cf*L-NPs kg^–1^ as compared to only *M. incognita*-infected plants (Figures 7A,B). The action of green synthesized Ag@*Cf*L-NPs, which boosts plant growth and development, was responsible for the decrease in gall number. Likewise, green synthesized Ag-NPs (which had not shown the phytotoxicity) have been reported to reduce the number of galls and suppressed the disease caused by root-knot nematode *M. javanica* in *Solanum melongena* (L.) plants (Abdellatif et al., 2016). In another study, green-synthesized Ag-NPs significantly reduced the disease attributes (galls number, egg masses, and root-knot index) in nematode infected *Solanum Lycopersicum* (L.) and improved the performance of plants (Hassan et al., 2016).

Furthermore, the incidence and severity of disease in tomato seedlings exposed to 50 and 100 mg kg^–1^ of synthesized Ag@*Cf*L-NPs, were evaluated. When compared to plants infected with *F. oxysporum* alone, the highest doses of Ag@*Cf*L-NPs (100 mg kg^–1^) demonstrated considerable antifungal efficacy against *F. oxysporum*, reducing disease incidence (55% reduction), and disease severity (43% reduction) (Figures 7C,D). The primary motivation for using nanomaterials in agriculture practices is to combat diseases, which frequently include compounds with antibacterial capabilities that target soil-borne pathogens. This might be due to a reduction in the ability of pathogenic fungi to produce enzymes and toxins, both of which are necessary for disease development. In field research, Giannousi et al. (2013) found that nano-Cu was more efficient than typical Cu-based formulations that suppressing the development and damage caused by *Phytophthora infestans* in tomato plants.

## Conclusion

In order to reduce the use of hazardous synthetic chemicals, efficient nano-pesticides that can be synthesized from natural metabolites of plants are critical. We examined and analyzed the anti-phytopathogenic efficacy of phytogenically synthesized Ag@*Cf*L-NPs against *R. solani* (fungi), *P. syringae* (bacteria), and *M. incognita* (root-knot nematode) which adversely affect the growth and yield of agronomically important edible crops. The green synthesized Ag@*Cf*L-NPs represented bactericidal, fungicidal as well as nematicidal activity. Increasing doses of Ag@*Cf*L-NPs inhibited the biofilm formation, impaired membrane integrity, and altered the morphology of *P. syringae*. Furthermore, anti-nematicidal potential of Ag@*Cf*L-NPs displayed as reduction in egg hatching and a considerable increased in larval motility of *M. incognita*. Moreover, when grown in the presence of synthesized Ag@*Cf*L-NPs, micro-morphological characteristics of all tested fungal hyphae were changed. Additionally, nano-pesticidal potential of Ag@*Cf*L-NPs was proved when applied to phytopathogens-challenged tomato crops cultivated in pots. The Ag@*Cf*L-NPs suppressed the disease severity and improved the plant growth, biomass, photosynthetic capacity, lycopene content, and antioxidative properties of tomatoes. As a result of the present study, it appears that Ag@*Cf*L-NPs with sufficient anti-phytopathogenic capability might be utilized as a safe and effective alternative to chemical control measures (synthetic pesticides) for managing phytopathogens that cause significant losses in agricultural productivity in different agro-climatic conditions. However, more research in this area is needed to assess the level of phytotoxicity under field conditions, which must be carried out before recommending the application of nano-based commercial products.

## Data availability statement

The original contributions presented in this study are included in the article/Supplementary material, further inquiries can be directed to the corresponding author.

## Author contributions

MD and LA: conceptualization and investigation. MD, MS, LA, and KR: methodology and writing – original draft preparation. AA and MA-D: validation. MS, MD, and US: formal analysis. MS, US, and YA-W: resources. AM: funding. MS, US, and SD: writing – review and editing and manuscript revision. All authors contributed to the article and approved the submitted version.
