# Supplementary material for: Nano-pesticidal potential of Cassia fistula (L.) leaf synthesized silver nanoparticles (Ag@CfL-NPs): Deciphering the phytopathogenic inhibition and growth augmentation in Solanum lycopersicum (L.)
Source: Front Microbiol. 2022 Aug 26;13:985852. doi: 10.3389/fmicb.2022.985852 (PMC9459237; doi:10.3389/fmicb.2022.985852)
Supplement: Supplementary file 1 [file Data_Sheet_1.docx]

**Electronic Supporting Information**

Nano-pesticidal potential of *Cassia fistula* (L.) leaf synthesized silver nanoparticles (Ag@*Cf*L-NPs): Deciphering the phytopathogenic inhibition and growth augmentation in *Solanum lycopersicum* (L.)

Mohammad Danish^1,^ *, Mohammad Shahid^2, 7^, Lukman Ahamad^3^, Kashif Raees^4^_,_ Ashraf Atef Hatamleh^5^, Munirah Abdullah Al-Dosary^5^, Abdullah Mohamed^6^, Yasmeen Abdulrhman Al-Wasel^5^, Udai B. Singh^7^

*^1^Section of Plant Pathology and Nematology, Department of Botany, Aligarh Muslim University, Aligarh-202002; Uttar Pradesh, India*

*^2^Department of Agricultural Microbiology, Faculty of Agricultural Sciences, Aligarh Muslim University, Aligarh-202002; Uttar Pradesh, India*

*^3^Section of Plant Pathology and Nematology, Department of Botany, Aligarh Muslim University, Aligarh-202002; Uttar Pradesh, India*

*^4^Department of Chemistry, Chandigarh University, Mohali, Punjab, India.*

*^5^Department of Botany and Microbiology, College of Science, King Saud University, P.O. Box 2455, Riyadh 11451, Saudi Arabia*

*^6^Research Centre, Future University in Egypt, New Cairo, 11745, Egypt*

*^7^Palnt-Microbe Interaction and Rhizosphere Biology Lab, ICAR-NBAIM, Mau, India*

**Correspondence to*:

Dr. Mohammad Danish

Section of Plant Pathology and Nematology,

Department of Botany

Faculty of Life Sciences,

Aligarh Muslim University, Aligarh, India

**E-mail:** [danish.botanica@gmail.com](mailto:danish.botanica@gmail.com)

**Supplementary Methods**

**S1.1 *Bacterial cell viability (CFU) assessment under* Ag@*Cf*L-NPs *stress***

To determine the potentiality of Ag@*Cf*L-NPs, the vitality of the test phytopathogenic bacterial isolate *Pseudomonas syringae* was determined by growing them in the presence of various doses (0-400 µgmL^-1^) Ag@*Cf*L-NPs. For CFU count, the 0.1 mL volume of 24 h grown pathogenic culture from each exposure concentration was spread plated on nutrient agar (NA) medium and incubated under the above-mentioned growth condition to count the viable cells. Number of colonies forming units (CFUs) per mL was converted to log_10_ CFU mL^-1^ and plotted as a function of pesticide concentration. The CFU was calculated as;

Colony forming unit (CFU) = Number of colonies × dilution factor

Volume plated

**S1.2 *Effect of* Ag@*Cf*L-NPs *on surface morphological changes in bacteria***

The interaction studies for observing morphological changes in the cells of *P. syringae* treated/untreated with Ag@*Cf*L-NPs was done by scanning electron microscopy (SEM; JEOL, Tokyo, Japan) as previously described by Ansari et al., (2014). In short, the samples containing bacterial cells (untreated/treated) were deposited on a Millipore filter (Millipore). A 10^6^ CFU/mL cell were treated with sub-MIC concentration of Ag@*Cf*L-NPs nanorod for 6 h in the presence of light at 37 ^0^C and centrifuged at 3000 rpm for 10 min. followed by three time washing with phosphate buffer saline (PBS) and pre-fixed with 2.5% glutaraldehyde for 1 h at 4 ^0^C. The pre fixed cells were washed twice followed by fixation with 1% osmium tetroxide for 1 h at room temperature. After three-time successive washing, samples were dehydrated with 30, 50, 70, 80, 90 and 100% of ethanol for 10 min each. The biomass of cell was then fixed and coated samples were observed under SEM.

**S1.3 *Permeability determination by confocal laser scanning microscopy (CLSM)***

In order to assess the effect of Ag@*Cf*L-NPs on cellular permeability of pathogenic isolate, confocal laser scanning microscopy (CLSM) was used (Shahid et al., 2021; Khan et al., 2020; Shahid et al., 2019; Shahid and Khan, 2018). For this, propidium iodide (PI) was used as a probe for the detection of cells with compromised cell membranes. Untreated and treated (with 100 µg mL^-1^ of Ag@*Cf*L-NPs) biofilm were washed three times with sterile PBS and stained with 100 μM PI for 15 min at room temperature in dark. The bacterial cells after incubation with PI were washed three times with PBS and mounted on a microscopic slide and then visualized using Leica TCS SPE, CLSM (Leica Microsystems, Germany).

***S1.4 Determination of EPS***

Bacterial cells were cultured in liquid medium treated with varied doses of Ag@*Cf*L-NPs to test the influence of NPs on extracellular polymeric substances (EPS) produced by bacterial isolates. The release of EPS in the presence of NPs was determined following the protocol as previously described by Khan et al., (2013). Briefly, supernatant from treated and untreated cells was separated at 8,000 rpm for 30 min. EPS in the supernatant was precipitated with three volumes of 95% ethanol followed by re-suspension in 200 μL of ultrapure water. The EPS was mixed with 1.2 mL mixture of ice-cold phenol (5%) and concentrated H_2_SO_4_ in the ratio of 1:5 (v/v). The absorbance of red color developed was quantified at 490 nm.

**S2 *Antioxidant enzymatic activities***

***S2.1 Determination of phenylalanine ammonia-lyase (PAL)***

For estimation of phenylalanine ammonia-lyase (PAL) [E.C. 4.1.3.5] 1.0 g leaf sample was homogenized in ice cold 0.1 M sodium borate buffer (2 ml; pH 7.0) having 1.4 mM mercapto-ethanol followed by centrifugation at 16,000 × *g* at 4 °C for 15 min. Reaction mixture containing enzyme extract (0.2 mL) was incubated with borate buffer (0.5 ml; pH 8.7) and distilled water (1.3 mL). Addition of 0.1 mM of l-phenylalanine (0.5 ml; pH 8.7) in each test tube incubated at 32 °C for 30 min initiated the reaction. The reaction was stopped by addition of 1.0 M trichloroacetic acid (0.5 mL). The absorbance was recorded at 290 nm and PAL activity was measured in terms of amount of t-cinnamic acid (t-CA) formed according to the method of Brueske (1980). It was expressed as μM TCA g^−1^ FW.

***S2.2 Determination of Polyphenol oxidase (PPO) [E.C. 1.14.18.1] activity***

The PPO activity was evaluated as described by Gauillard et al. (1973) with catechol as substrate for PPO. The leaf samples (0.1 g) were homogenized in ice cold 0.1 M phosphate buffer (5.0 mL; pH 6.5). The homogenate was centrifuged at 16,000 × *g* for 30 min at 4 °C. The supernatant obtained was used directly for the enzyme assay. The reaction mixture contained 0.01 M catechol (0.4 ml) in 0.1 M sodium phosphate buffer (3.0 ml; pH 6.5) and enzyme extract (0.4 mL). The absorbance at 495 nm was recorded at 30 s interval up to 3 min. PPO activity was expressed as change in OD min^−1^ g^−1^ FW.

**Supplementary Tables**

**Table S1:** One-way analysis of variance for Ag@*Cf*L-NPs on mycelia growth and inhibition of *R. solani*

| Nanoparticles | Df | Sum of  Square | Mean of  Square | *F*-value | Pr(>F) |
| --- | --- | --- | --- | --- | --- |
| Mycelia growth | 4 | 37.0 | 9.249 | 71.15 | 2.63e-07 *** |
| Residuals | 10 | 1.3 | 0.130 | - | - |
| Inhibition | 3 | 1556.4 | 518.8 | 94.56 | 1.38e-06 *** |
| Residuals | 8 | 43.9 | 5.5 | - | - |

*** = values are significantly different at p= 0.001; ns= values are not significantly different

**Table S2:** Effects of green synthesized silver nanoparticles (Ag@*Cf*L-NPs) on the hatching and mortality of *M. incognita*

| Nanoparticles | Concentration | Number of juveniles of *M. incognita* after (Hatching)  48 h | % Inhibition | Number of juveniles (Mortality) after  48 h | % Inhibition |
| --- | --- | --- | --- | --- | --- |
| Ag-NPs | 25 mg/L | 95b | 56.0 | 53a | 55.7 |
|  | 50 mg/L | 76c | 64.8 | 49b | 57.89 |
|  | 100 mg/L | 61d | 71.75 | 37c | 60.65 |
|  | 200 mg /L | 38e | 82.40 | 25d | 65.78 |
|  | 400 mg /L | 14 | 90.45 | 14.4 | 78.3 |
| Control | DW | 216a | 0.00 | 10e | 3.62 |
| L.S.D (p≤0.05) | - | **11.03** |  | **6.80** |  |

Data represents above are mean of three replicates (n = 3). Duncan’s new multiple range tests indicate data with different letters are significantly different at p≤0.05.

**Table S3:** One-way analysis of variance for Ag@*Cf*L-NPs on hatching and mortality of *M. incognita*

| Nanoparticles | Df | Sum of  Square | Mean of  Square | *F*-value | Pr(>F) |
| --- | --- | --- | --- | --- | --- |
| egg hatching | 4 | 58148 | 14537 | 395 | 5.87e-11 *** |
| Residuals | 10 | 368 | 37 | - | - |
| Larval mortality | 4 | 3380 | 845.1 | 60.36 | 5.77e-07 *** |
| Residuals | 10 | 140 | 14.0 | - | - |

*** = values are significantly different at p= 0.001; ns= values are not significantly different
